# Point-of-care smell testing for evaluation of olfactory function: a narrative review of clinical utility

**DOI:** 10.3389/falgy.2026.1882615

**Published:** 2026-06-26

**Authors:** Akshita Joshi, Haley Ogbemudia, Cailin Cruess, Alicia A. Livinski, Rosangele Hall, Ethan Roback, Jasmine Jain, Aarti Sharma, Prestina Smith-Davidson, Mei Xu, Valerie Asher, Paule V. Joseph, Joshua M. Levy

**Affiliations:** 1 Division of Intramural Research, National Institute on Deafness and Other Communication Disorders (NIDCD), National Institutes of Health, Bethesda, MD, United States; 2 National Smell and Taste Center, Division of Intramural Research, National Institutes of Health, Bethesda, MD, United States; 3 University of Maryland School of Medicine, Baltimore, MD, United States; 4 National Institutes of Health Library, Office of Research Services, National Institutes of Health, Bethesda, MD, United States; 5 Section of Sensory Science, and Metabolism, National Institute on Alcohol Abuse and Alcoholism (NIAAA), National Institutes of Health, Bethesda, MD, United States; 6 Emory University School of Medicine, Atlanta, Georgia, United States; 7 Concord Fragrances LLC, Bethesda, MD, United States

**Keywords:** clinical utility, cultural adaptation, olfactory dysfunction, point-of-care testing, psychophysical olfactory assessment

## Abstract

**Importance:**

Olfactory dysfunction (OD) is increasingly recognized as a clinically relevant biomarker for a wide range of health conditions, including neurodegenerative diseases, respiratory infections such as COVID-19, psychiatric disorders, and age-related sensory decline. Despite its diagnostic value, olfactory testing remains underutilized in routine clinical care due to challenges related to test accessibility, cultural adaptability, cost and practical implementation.

**Observations:**

This narrative review provides a clinically focused synthesis of olfactory tests suitable for point-of-care (POC), focusing on their diagnostic accuracy, administration methods, user-friendliness, and applicability across diverse clinical populations. Commonly used tests such as the University of Pennsylvania Smell Identification Test (UPSIT®) and Sniffin' Sticks® are discussed alongside emerging rapid screening tools, pediatric assessments, and culturally adapted tests. Attention is given to distinguishing screening tools from comprehensive diagnostic batteries and to the role of point-of-care (POC) testing in primary care, neurology, and otorhinolaryngology settings. Practical considerations including assessment time, workflow feasibility, and clinical decision pathways are also examined. This review emphasizes the translation of available psychophysical tests into a practical POC framework for real-world clinical use.

**Conclusions and relevance:**

Available olfactory tests vary substantially in diagnostic accuracy, feasibility, and clinical applicability, highlighting the importance of selecting tests based on clinical indication and practice setting. Brief POC tests are best positioned as screening tools, while comprehensive batteries remain necessary when diagnostic precision is required. The main contribution of this review is a clinically oriented framework that distinguishes screening from diagnostic testing and aligns test selection with adult and pediatric clinical scenarios, workflow constraints, and referral needs. A tiered clinical approach using rapid screening followed by targeted comprehensive testing may represent the most practical, cost and time effective strategy for integrating olfactory assessment into routine care. Broader implementation of clinically feasible olfactory testing may improve early disease detection, support clinical decision making, and enhance patient care across conditions associated with OD.

## 1 Introduction

The sense of smell is a noninvasive biomarker for brain health and human disease. Olfactory dysfunction (OD), an altered sense of smell, is associated with normal aging (1) and more than 130 medical conditions (2), including viral respiratory infection and neurodegenerative disease, where OD may be an early or a presenting symptom (3–5). While the prevalence of OD is difficult to measure, prior to the COVID-19 pandemic, OD (including anosmia and hyposmia) affected an estimated 20% of the general population (6). During the SARS-CoV-2 pandemic, acquired OD increased sharply, with loss of smell presenting as a primary symptom in both acute and long-COVID (7, 8). During the early waves of the SARS-CoV-2 pandemic, acute COVID-19 had a particularly strong association with OD (9), and several studies reporting a higher positive predictive value for smell loss than for other symptoms such as fever (7, 8).

OD is an important biomarker in several neurodegenerative diseases, often preceding other focal deficits by years in conditions such as Alzheimer's Disease (AD) and Parkinson's Disease (PD) (10–12). Early identification of OD may therefore facilitate earlier diagnosis and treatment initiation for chronic disease, improving patient outcomes while increasing clinical interest in olfactory testing (13).

Despite this increased interest, consensus is lacking on the most appropriate point-of-care (POC) screening test. The recent proliferation of olfactory assessment tools further complicates test selection, particularly because available tests differ in administration time, diagnostic depth, cultural familiarity, pediatric suitability, cost, and workflow requirements. Prior reviews have summarized olfactory dysfunction, psychophysical testing methods, or disease-specific applications of olfactory assessment; however, fewer have focused specifically on how clinicians should select and implement olfactory tests in real-world POC settings. The novelty of this review lies in its clinically oriented synthesis of available olfactory tests into practical adult and pediatric implementation frameworks. Specifically, this review distinguishes rapid screening tools from comprehensive diagnostic batteries, integrates cultural and developmental considerations, and proposes a tiered approach to guide test selection based on clinical indication, feasibility, and need for specialty referral.

## 2 Methods

This narrative review provides a structured overview of currently available clinical olfactory tests and their diagnostic characteristics. This review was exempt from Institutional Review Board oversight.

A literature search was performed in PubMed (U.S. National Library of Medicine). The search strategy included keywords related to olfactory testing. Searches were limited to articles published in English between January 2014 and December 2024. The complete search strategy is provided in Supplementary File 1. Animal studies and specific publication types as specified in the eligibility criteria were excluded using search strategies. Additionally, the bibliographies of the articles included after full text screening and relevant reviews were scanned.

Studies were included if they met the following eligibility criteria: (1) population: Adult and pediatric patients with suspected or diagnosed smell disorders; (2) intervention: Diagnostic tests for smell function, including psychophysical, chemical assessments; (3) outcomes: Measures of diagnostic accuracy, including sensitivity, specificity, positive predictive value (PPV), and negative predictive value (NPV); and (4) study design: Observational studies (cross-sectional and cohort studies) as well as randomized controlled trials (RCTs) that provided data on the diagnostic performance of smell tests. Studies were excluded if they did not include primary data on diagnostic performance, were review articles of any type, case reports, or focused exclusively on animal models.

Screening was completed at two stages using Covidence (Veritas Health Innovation, Melbourne, Australia). At both stages, two reviewers independently and in duplicate screened each record. At stage one, titles and abstracts of all unique records retrieved from the search were screened. At stage two, full texts of studies included after abstract screening were reviewed. Discrepancies were resolved through discussion between reviewers and senior authors.

Collected data included (1) study characteristics: author, year of publication, study design, country, and sample size; (2) population characteristics: patient demographics, underlying conditions, and type of chemosensory dysfunction; (3) test details: type of test (psychophysical, chemical), method of administration, and diagnostic thresholds; and (4) diagnostic performance outcomes: sensitivity, specificity, PPV, NPV, and area under the receiver operating characteristic (ROC) curve when reported.

Given the heterogeneity of included studies and the descriptive aims of this review, data on key test characteristics, including ease of use, feasibility in varied populations, and cultural adaptability were narratively summarized and synthesized. No formal quality assessment or meta-analysis was performed as the objective was to provide a comprehensive descriptive synthesis of the existing evidence.

The database searches yielded 2,562 articles, of which 24 were duplicates, leaving 2,538 for title and abstract review. 562 studies were screened at the full text stage, resulting in the exclusion of 386 and inclusion of 176 articles.

### 2.1 Characteristics of included studies and tests

The literature reviewed represents a broad body of publications addressing clinical olfactory testing. Key and representative studies were discussed to provide a clinically oriented overview of commonly used tests and their diagnostic characteristics. These studies included validation studies, clinical trials, and observational investigations across diverse clinical populations, including neurological disorders, psychiatric conditions, infectious diseases, and idiopathic olfactory dysfunction.

Key study characteristics and test features are summarized in Table 1. The table is structured to facilitate comparison of olfactory tests by patient population, psychophysical domain assessed, threshold, discrimination, and/or identification, primary use, MCID where available, and validated languages. The identified tests represent a range of administration formats, including scratch-and-sniff tests (e.g., UPSIT®, SCENTinel®), pen-based odor dispensing systems (e.g., Sniffin' Sticks®), and emerging digital or remote testing platforms (e.g., u-Smell-It™) (see Supplementary File 2 for a catalog of clinical olfactory tests).

**Table 1:** Characteristics of olfactory tests.

Patient population	Test name	T	D	I	Area of primary use	MCID	Validated languages
Adults and Children	Ascending Methods of Limits (AML)	X			Clinical	N/A	Eng
	Sniff Magnitude Test (SMT)	X			Clinical	1.0	Eng
	Snap & Sniff®	X	X		Clinical	N/A	Eng
	Digital Olfactory Testing System (DOTS)		X		Research	4.0	Eng
	Smell Identification Test (SIT™)			X	Clinical	1.0	Eng, Spa, Chi, Jpn, Fre, Ger, Ita, Ara, Dut, Por, Pol, Swe, and Tur
Adults	1967 Olfactory Test by Henkin	X	X	X	Clinical	N/A	Eng
	Olfactory Perception Threshold Test (OPTT)	X			Clinical	1.0	Eng
	Sniff Bubble			X	Clinical	N/A	Eng
	Asian T and T Test (T&T Test)	X	X	X	Clinical	3.4	Jpn and Chi
	Japanese T&T Olfactometer	X		X	Clinical	N/A	Jpn
	Odor Identification Test			X	Clinical	1.0	Eng, Spa, Fre, Ger, Ita, Chi, Kor, and Jpn
	Olfactory Test	X	X	X	Clinical	1.0	Eng, Fre, Ger, and Spa
	Smell Awareness Test	X	X	X	Clinical	N/A	Eng, Fre, Ger, Spa, Chi, Kor, and Ita
	University of Pennsylvania Smell Identification Test (UPSIT®)	X	X	X	Clinical	4.0	Eng, Spa, Fre, Ger, Ita, Chi, Kor, and Jpn
	Japanese Open Essence		X		Clinical	N/A	Jpn
	Connecticut Chemosensory Clinical Research Center (CCCRC) Test	X		X	Research	N/A	Eng and Por
	Quick Olfactory Sniffin’ Sticks Test (Q-Sticks)			X	Clinical	N/A	Eng
	Cross-Cultural Smell Identification Test (CCSIT)			X	Clinical	N/A	Eng
	Alcohol Sniff Test (AST)	X			Clinical	N/A	Eng
	Sniffin’ Sticks	X	X	X	Research	5.5	Eng, May, and Spa
	Affective Importance of Odor Scale (AIO)			X	Research	N/A	Eng
	Smell Diskettes Olfaction Test (SDOT)	X	X	X	Clinical	N/A	Eng
	Retronasal Olfactory Test			X	Clinical	N/A	Eng
	European Test of Olfactory Capabilities	X	X	X	Clinical	N/A	Fre, Ger, Swe, and Dut
	OSIT-Japanese Odor Stick Test			X	Clinical	N/A	Jpn
	Barcelona Smell Test-24 (BAST-24)		X		Clinical	N/A	Spa
	The Questionnaire of Olfactory Disorders (QOD)				Clinical	5.2	Chi, Eng, and Kor
	Sino-Nasal Outcome Test (SNOT-22)	X			Clinical	1.8	Eng, Spa, Fre, Ara, Ger, and Ita
	Odor Awareness Scale (OAS)	X			Clinical	N/A	Eng
	Candy Smell Test (CST)	X		X	Clinical	N/A	Eng
	Pocket Smell Test® (PST)	X		X	Clinical	N/A	Eng, Ger, Spa, Fre, and Por
	NIH Odor Identification Toolbox			X	Clinical	N/A	Eng and Spa
	Jet Stream Olfactometry (JSO)		X	X	Clinical	N/A	Jpn
	Self-administered Olfactory Testing System	X		X	Clinical	1.0	Eng
	Smell-Sensitivity and Smell-Resolution Odor Test		X	X	Clinical	7.0 for Smell-S, 8.5 for Smell-R	Eng
	Indian Smell Test in COVID-19 by AIIMS	X		X	Clinical	N/A	Hin
	Adaptive Olfactory Measure of Threshold (ArOMa-T)	X	X	X	Clinical	1.0	Eng
	SCENTinel®	X		X	Clinical	1.0	Eng
Children	Pediatric Barcelona Olfactory Test-6 (pBOT-6)	X		X	Clinical	N/A	Spa
	Sniffin’ Kids Test	X	X	X	Clinical	3.0	Eng
	Pediatric Smell Wheel™	X		X	Clinical	N/A	Eng
	Universal Sniff Test (U-Sniff)			X	Clinical	N/A	Eng

Ara, Arabic; Chi, Chinese; D, Discrimination; Dut, Dutch; Eng, English; Fre, French; Ger, German; Hin, Hindi; I, Identification; Ita, Italian; Jpn, Japanese; Kor, Korean; May, Malay; MCID; Minimal Clinically Important Difference; Pol, Polish; Por, Portuguese; Spa, Spanish; Swe, Swedish; T, Threshold; Tur, Turkish.

## 3 Overview of olfactory testing

Psychophysical olfactory tests are widely used in clinical practice. These tests, which measure an individual's ability to detect odors at low concentrations (threshold), distinguish between different odors (discrimination), or correctly recognize and name odors (identification) (14), rely on patient participation and self-report, making them patient-dependent rather than fully objective. However, they are currently the most feasible and accessible option for routine assessment. Truly objective olfactory tests are not currently validated for clinical use.

Tests in both adults and children must account for cognitive development, cultural familiarity with odorants, and attention span, all of which influence accuracy and feasibility. Below is a summary of validated olfactory tests available for assessment in adults and pediatric populations.

### 3.1 Olfactory testing in adults

Adult psychophysical olfactory assessments include tools designed to evaluate odor threshold, discrimination, and identification. The Sniffin' Sticks® battery (15) is among the most extensively validated tools. The full threshold-discrimination-identification (TDI) assessment takes approximately 45 min to complete and requires trained staff to administer, while shorter versions such as the 12-item Sniffin' Sticks (SS-12) offer faster screening with reported sensitivity of 93.4% and specificity of 68.2% in a COVID-19-related olfactory dysfunction screening population (16). The 32-item extended identification version demonstrates strong reliability (*r* = 0.76), with slightly higher stability in controls (*r* = 0.80) than in clinical populations (*r* = 0.67) (17) and self-administered adaptations show moderate to excellent test–retest reliability (0.51–0.93) (18).

The University of Pennsylvania Smell Identification Test (UPSIT®) (19), is another widely used self-adminstered tool. This 40-item scratch-and-sniff test takes approximately 10–15 min (20) and demonstrates strong diagnostic performance in neurodegenerative cohorts with reported sensitivity and specificity of 82% and 66% and a high test-retest reliability (0.94) in studies evaluating olfactory identification deficits in Parkinsonian or related neurodegenerative populations (21, 22). Abbreviated versions such as NHANES Pocket Smell Test (N-PST®), the 12-item Brief Smell Identification Test (B-SIT®) with test-retest reliability of 0.81, and the 3-item PST® improve feasibility. The PST® takes 30 s to administer and yields 95% accuracy in Parkinson's and Alzheimer's patients (23, 24) and 83% accuracy in chronic rhinosinusitis (25). A newer 4-item UPSIT-derived screening test detected hyposmia in Parkinson's disease with 81% sensitivity, outperforming the PST® (26). The Connecticut Chemosensory Clinical Research Center (CCRC) test remains a widely used low-cost option combining threshold and identification testing and can distinguish unilateral from bilateral (27).

The COVID-19 pandemic accelerated development of rapid screening tools. Tests such as the Quick Smell Identification Test (28), the Singapore Smell and Taste Test (SSTT) (29), the Indian Smell Test (30), and U-Smell-It™ demonstrated variable performance, with U-Smell-It™ showing 89.2% sensitivity but modest specificity (31). The SS-12 was also validated for COVID-19 screening (32), including disposable filter-paper formats (33). SCENTinel® emerged as a particularly promising rapid screening test, demonstrating 72% sensitivity and 94% specificity COVID-19-related smell-loss screening, with additional utility reported in acute and long COVID-19 cohorts (34, 35). In a post-viral cohort, the Alcohol Sniff Test provided an inexpensive alternative with reported sensitivity of 88% (36), although the PST® demonstrated reduced diagnostic accuracy in detecting COVID-related olfactory loss (37).

Newer threshold-focused tools offer practical advantages for routine adult testing. The Adaptive Olfactory Measure of Threshold (ArOMa-T) provides threshold estimates in under three minutes without the need for trained administrators (38). The Snap & Sniff® test provides a rapid 15-minute threshold assessment with strong reliability (*r* = 0.84) and high correlations with UPSIT® and traditional threshold tests (*r* > 0.65–0.88) (39). Together, these tools provide flexible options that balance diagnostic depth with feasibility in POC settings.

When selecting among tests, clinicians must balance comprehensiveness with feasibility. Comprehensive batteries such as Sniffin' Sticks® provide multidimensional diagnostic information, whereas abbreviated identification tests such as B-SIT® and SCENTinel® provide rapid screening with less pathophysiological detail. Identification tests may be preferable for neurological screening, whereas threshold testing may better characterize sinonasal disease. Thus, test selection should be guided by the clinical question rather than convenience alone.

#### 3.1.1 POC olfactory testing in adults

Psychophysical olfaction tests can be broadly categorized into screening and diagnostic tools. Screening tests such as SCENTinel®, PST®, B-SIT®, and SS-12 prioritize speed and feasibility, whereas diagnostic tools such as the Sniffin' Sticks TDI battery and UPSIT® provide more comprehensive characterization when diagnostic precision is required. Rapid threshold tools such as ArOMa-T and Snap & Sniff® (40) are particularly suited for POC environments due to short administration times.

POC testing requires balancing diagnostic value with clinical practicality. Test selection should align with the clinical question and the specific needs of each specialty. In neurology, identification-focused tests such as UPSIT® support early detection of neurodegenerative disease (19, 41), including Alzheimer's disease (42), Parkinson's disease (43), and Multiple Sclerosis (44). The full Sniffin’ Sticks® battery may also be useful when more comprehensive evaluation of central olfactory dysfunction is required, while identification and discrimination subtests may assist in rapid clinical assessment.

In ENT and allergy settings, where peripheral dysfunction is common, threshold testing such as the Sniffin' Sticks® threshold subtest and rapid threshold tools such as ArOMa-T may be useful for evaluating chronic rhinosinusitis and nasal polyposis (5, 45). The CCRC test and full Sniffin' Sticks® battery remain useful when detailed characterization is required and may guide treatment planning (46, 47).

Post-viral olfactory dysfunction has increased reliance on POC screening tools such as SCENTinel® and SS-12 to monitor recovery, with simpler approaches such as the Alcohol Sniff Test offering inexpensive screening options (35, 48). In contrast, head trauma and congenital anosmia, often require more comprehensive testing such as full Sniffin' Sticks® or UPSIT® for diagnostic clarification (4, 49, 50).

In primary care, time and resource limitations favor brief tests such as SS-12, UPSIT® short forms, B-SIT®, PST®, and SCENTinel®, which support rapid identification of olfactory dysfunction and facilitate referral decisions. Increasing validation of self-administered and culturally adapted tools has also expanded the feasibility of home-based testing (51, 52).

### 3.2 Olfactory testing in children

Pediatric olfactory assessment requires developmentally appropriate tests using familiar odorants. For children aged 3–7 years, the U-Sniff® test is the most widely validated option. This 12-odorant identification test incorporates pictorial cues and has been validated in multiple countries with test-retest reliability ranging from 0.61 to 0.80 (53, 54). A shorter 5-item version (qU-Sniff), developed for ages 6–17 years, demonstrated a test-retest reliability of 0.626 (55).

For ages 6–17 years, the Sniffin' Kids Test, adapted from the Sniffin' Sticks® battery, provides age-adjusted identification tool uses 14 familiar odorants. Although reliability is modest (0.44), it remains one of the few structured pediatric smell tests (56). Cultural adaptations (Sniffin' Kids-PT) have further improved applicability across populations (57).

The UPSIT-Children's Version allows more comprehensive testing in children aged five years and older. However, shorter tools such as the Sniffin' Sticks-12 are often preferred clinically due to shorter testing time and reduced olfactory fatigue (58). The Pediatric Smell Wheel™ provides an engaging alternative using pictorial odor matching, with moderate reliability demonstrated in Brazilian cohorts (59). The pBOT-6 combines identification and threshold testing and shows high diagnostic accuracy (sensitivity and specificity of 96.6% and 100%), although its use remains largely research-focused (60).

Remote olfactory testing has also expanded to include children. During the COVID-19 pandemic, the German Chemosensory Pleasure Scale for Children (CPS-C(de)) emerged as a home-based option, showing comparable results to U-Sniff with minimal bias. These developments highlight the increased potential for incorporating olfactory assessment into both clinical and remote pediatric olfactory assessment.

#### 3.2.1 POC olfactory testing in children

POC olfactory testing in children requires brief, reliable, and developmentally appropriate tools. For younger children (3–7 years), the U-Sniff® remains the most practical POC option due to simplicity, pictorial designs, and strong cross-cultural validation. Reported sensitivity and specificity range from 79%–93% to 88%–95%, respectively, with testing reliability beginning at age four (54, 61). For older children (6–17 years), Sniffin' Kids provides efficient odor identification testing suitable for both clinical and research settings (56, 57).

Additional pediatric POC options include the UPSIT-Children's Version and the Smell Wheel™, while abbreviated tests such as SS-12 are often preferred when rapid screening is needed in older children (58, 62). While the pBOT-6 demonstrates exceptional diagnostic accuracy (96.6% sensitivity, 100% specificity), limited validation outside a Spanish cohort restricts routine POC use (60).

In clinical practice, U-Sniff® and Sniffin’ Kids are most commonly used for general screening, post-viral olfactory dysfunction, congenital conditions, as they provide a balance between feasibility and diagnostic value. Ultimately, the choice of a pediatric POC test depends on developmental level, clinical context, and time constraints. The use of validated pediatric-specific tools will allow clinicians to identify olfactory dysfunction in children and determine whether specialty referral or additional testing is warranted.

### 3.3 Emerging & specialized olfactory tests

Newer olfactory assessment tools use digital, automated, and self-administered platforms to improve accessibility and support large-scale or remote screening. The Noar Multiscent-20 test is a tablet-based odor identification system integrating a digital interface with an automated odor-release unit, enabling independent administration and automatic data recording. Compared to the 40-item UPSIT®, it demonstrated strong test–retest reliability (0.820) (63). Similarly, the Digital Olfactory Testing System (DOTS), which includes an odor-delivery device (DOTS-ODD) paired with a mobile application (DOTS-APP), and incorporates UPSIT® scoring, exhibits a strong correlation with standard UPSIT® (*r* = 0.714) and test-retest reliability of 0.807 (64, 65). Although not yet widely used, DOTS represents a promising approach for home-based olfactory screening. Another emerging platform, the Digital Scent Device 20 (DSD-20), delivers a set of 20 universal odorants and has shown a strong correlation with Sniffin' Sticks® (*r* = 0.80) and high reliability (0.88) in European validation studies (66).

Digital tools offer scalable solutions for rapid, standardized testing outside of traditional clinical settings and may support population-level screening, remote monitoring, and longitudinal tracking. However, broader implementation will require further standardization of odor delivery, and comparison with established psychophysical tests. As these digital tools evolve, they have the potential to expand access to olfactory testing.

### 3.4 Cultural adaptations

Cultural adaptation of olfactory tests ensures valid results across diverse populations by using familiar odorants. Region-specific tools address differences in odor familiarity, semantic associations, and linguistic context. For Spanish-speaking populations, the Barcelona Smell Test 24 (BAST-24) and its shorter BOT-8 version demonstrate excellent reliability (67). The Iran Smell Identification Test (ISIT), with 92% reliability (68), was developed after UPSIT® was shown to include odors unfamiliar to Iranian cohorts (69). A Chinese adaptation (UPSIT-TC) demonstrated strong reliability in Taiwanese patients with chronic rhinosinusitis and Parkinson's disease (70). A Brazilian UPSIT® similarly showed strong cultural fit (71), and adaptations also exist for Saudi Arabian and Turkish populations (72, 73).

Sniffin' Sticks® has been adapted across Portuguese, Spanish, Danish, Iranian, South Kivu, and Malay populations (74–82). Additional adaptations include a South Korean smell identification test tailored specifically for Korean odor familiarity (83).

The CCRC test has been modified for use in Brazil and Turkey, where culturally relevant odorants improved reliability and diagnostic accuracy (46, 84, 85).Other self-administered tool, such as Smell-S and Smell-R, have also been developed to reduce odor-specific cultural bias and demonstrated lower cross-cultural variability between North American and Taiwanese subjects (86). Together, these culturally adapted tests highlight the importance of regional validation to ensure diagnostic precision across diverse populations.

### 3.5 Patient reported outcome measures (PROMS)

PROMS quantify the subjective impact of OD and complement psychophysical testing at the POC. They assess symptom severity, quality-of-life effects, and longitudinal change, though correlation with objective measures are modest (87). Available PROMs include the International Survey for Clinical Assessment of Smell (50, 88) and multiple culturally adapted versions of the Questionnaire of Olfactory Disorders (89–97). Other tools such as the Self-MOQ (98–101) the QOD- (102–104). While validation is limited to patients with chronic rhinosinusitis, the 22-item Sinonasal Outcomes Test (SNOT-22) (105) further characterizes the functional and emotional burden of symptoms related to chronic sinusitis, including sense of smell and taste. PROM data are most valuable when paired with objective olfactory tests, ensuring a more holistic evaluation in both clinical care and research.

## 4 Clinical considerations

Effective POC olfactory testing requires attention to patient factors, test features, and the testing environment. Threshold-based tools such as the Sniffin' Sticks® threshold subtest are useful for detecting early or peripheral impairments (45), while identification-based tools like UPSIT® help evaluate central dysfunction, including neurodegenerative disease (14). Odor-delivery methods, such as jet-stream olfactometry (106), glass vials, or pen-based formats, can influence stimulus perceived intensity and consistency (107). Adequate intervals between odors reduce adaptation effects, particularly in patients with olfactory loss (108), and feedback should be avoided to minimize learning effects.

Environmental factors also influence test performance. Proper ventilation prevents residual odor contamination (109), while ambient scents from disinfectants, food, or perfumes can distort odor thresholds (110, 111). Noise, humidity, temperature (112), and air pollution (113) may further affect attention and perception. Because time and resources in primary care often preclude the use of lengthy batteries, shorter tools such as SCENTinel®, PST®, and abbreviated Sniffin' Sticks® formats are often preferred despite slightly lower sensitivity. Digital tools such as DOTS and Noar Multiscent-20 (114) may further improve feasibility but require further validation.

Although evidence directly linking POC testing to improved outcomes remains limited, available data suggests it can influence referral patterns, diagnostic evaluation, and monitoring of recovery after infection or trauma. Current evidence therefore supports POC testing primarily as a screening tool rather than a definitive diagnostic method.

## 5 Clinical implications and conclusion

Quantitative olfactory testing has advanced considerably, with UPSIT® and Sniffin' Sticks® established as core tools for assessing threshold, discrimination, and identification in clinical settings. Psychophysical tests provide standardized but patient-dependent measures of olfactory performance. Importantly, abbreviated POC tests should be considered screening tools rather than substitutes for comprehensive olfactory evaluation. Full batteries such as the Sniffin' Sticks provide multidimensional diagnostic information that brief identification tests do not provide, and may help differentiate peripheral dysfunction (e.g., CRS) from central causes such as neurodegenerative disease. However, the time required for comprehensive batteries limits their routine use in time-constrained clinical settings.

Experience following the COVID-19 pandemic has further emphasized the need for rapid, reliable, and scalable tests capable of distinguishing quantitative dysfunction from qualitative dysfunction and supporting remote or POC use. From a clinical practice perspective, a structured testing strategy may provide the most practical implementation framework. In primary care, rapid screening tools such as SCENTinel® or B-SIT® may be used initially, followed by referral for comprehensive testing when abnormalities are detected. In ENT practice, threshold testing may help identify peripheral causes, while neurology clinics may prioritize identification testing for neurodegenerative screening. Such stratified use of olfactory testing may improve diagnostic efficiency while minimizing unnecessary resource utilization.

The tiered approach proposed here is derived from the narrative synthesis of the included literature rather than from a single prospectively validated clinical pathway. Across the reviewed studies, a consistent practical distinction emerges between brief screening tools, which prioritize speed, ease of administration, and workflow feasibility, and comprehensive olfactory batteries, which provide greater diagnostic detail but require more time, resources, and trained administration. Therefore, the proposed framework should be interpreted as an evidence-informed clinical implementation model designed to guide test selection according to clinical indication, patient population, and practice setting. Prospective studies will be needed to formally validate whether such tiered pathways improve diagnostic efficiency, referral patterns, patient outcomes, or cost-effectiveness in routine care.

**Figure 1:**
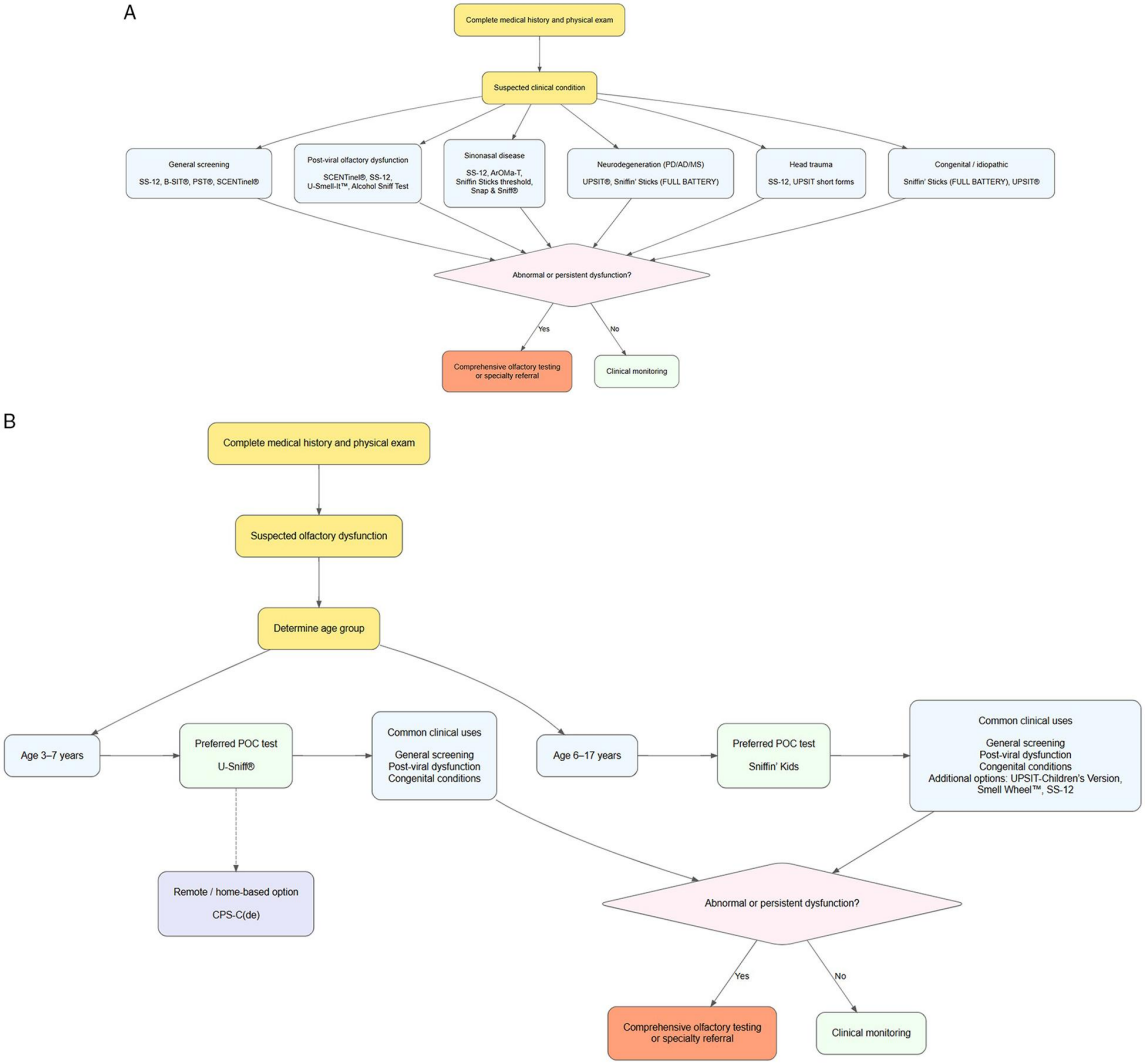
Practical implementation frameworks for point-of-care olfactory screening in adult and pediatric populations. **(A)** Adult POC olfactory screening clinical decision framework. Following medical history and physical examination, suspected clinical conditions can guide selection of appropriate screening tools. Brief tests such as SS-12, B-SIT®, PST®, SCENTinel®, U-Smell-It™, Alcohol Sniff Test, ArOMa-T, Snap & Sniff®, and UPSIT® short forms may be considered depending on clinical indication. Comprehensive testing or specialty referral should be considered when dysfunction is abnormal, persistent, or diagnostically unclear. **(B)** Pediatric POC olfactory screening clinical decision framework. Following medical history and physical examination, age group is a primary determinant of test selection. U-Sniff® is preferred for children aged 3–7 years, whereas Sniffin’ Kids and selected additional tools, including UPSIT-Children's Version, Smell Wheel™, and SS-12, may be considered for children aged 6–17 years. Persistent or abnormal dysfunction should prompt comprehensive olfactory testing or specialty referral.

A key contribution of this review is the translation of available evidence on olfactory test performance and feasibility into practical implementation frameworks for POC olfactory testing in adult and pediatric populations (Figures 1A,B). Rather than presenting olfactory tests as interchangeable tools, these frameworks emphasize that test selection should be guided by the clinical question, patient age, suspected etiology, and available clinical resources. In adults, the framework organizes test selection by suspected clinical indication, whereas in children, age and developmental suitability are emphasized as primary considerations. Table 2 complements these frameworks by summarizing suggested POC screening tests across common clinical settings, along with relative cost and workflow feasibility. Together, Figures 1A,B and Table 2 are intended to support practical clinical test selection while recognizing that abnormal, persistent, or diagnostically unclear findings may require comprehensive olfactory testing or specialty referral.

**Table 2:** Suggested point-of-care (POC) olfactory screening tests across clinical settings in adults and children.

Clinical setting	Adults: suggested POC screening tests	Children: suggested POC screening tests	Approximate testing time	Relative implementation feasibility*
General screening	SS-12, B-SIT®, PST®, SCENTinel®	U-Sniff®, Sniffin’ Kids	≤10 min	High- brief screening
Post-viral OD	SCENTinel®, SS-12, U-Smell-It™, Alcohol Sniff Test	U-Sniff®, Sniffin’ Kids	<15 min	High- brief screening
Sinonasal disease	SS-12, ArOMa-T, Snap & Sniff®, Sniffin’ Sticks threshold	U-Sniff®, Sniffin’ Kids	∼20 min	Moderate- threshold focused setup
Neurodegeneration (PD/AD/MS)	UPSIT®, Sniffin’ Sticks (full battery)	Sniffin’ Kids	∼45 min	Lower- comprehensive testing
Head trauma	SS-12, UPSIT short forms	U-Sniff®, Sniffin’ Kids	<15 min	High- brief screening
Congenital/idiopathic	Sniffin’ Sticks (full battery), UPSIT®	U-Sniff®, Sniffin’ Kids	∼20–45 min	Lower/moderate- comprehensive testing

*Relative implementation feasibility was assigned by the authors based on the narrative synthesis of the publications cited throughout this review. The classification considered approximate testing time, material or proprietary burden, consumable requirements, training requirements, setup complexity, scoring burden, and ease of integration into routine clinical workflow. These categories are intended as pragmatic clinical descriptors rather than validated feasibility scores or formal cost-effectiveness rankings. **High feasibility** reflects brief screening approaches with lower material/setup burden, minimal training requirements, and easier integration into routine workflow; **moderate feasibility** reflects intermediate testing time and/or setup burden, often involving standardized kits, multiple test components, threshold-focused setup, or some trained oversight; and **lower feasibility** reflects comprehensive or proprietary testing approaches that require longer administration time, greater material/setup burden, trained personnel, and/or more complex scoring or administration.

Cost and workflow feasibility therefore remain key determinants of POC adoption. Brief screening tests such as SCENTinel®, PST®, and SS-12 are relatively inexpensive, require minimal training, and can be completed rapidly, making them suitable for frontline clinical settings. In contrast, comprehensive batteries such as Sniffin' Sticks®, while diagnostically informative, require more time and resources. A cost-conscious clinical model may therefore involve rapid screening followed by targeted comprehensive testing when clinically indicated.

In summary, integration of culturally adaptable tools, validated rapid screening assessments, and emerging digital technologies within structured clinical pathways may improve the diagnostic precision, accessibility, and real-world clinical utility of olfactory testing. From a health-system perspective, using low-cost screening tools followed by targeted comprehensive testing may represent a practical and resource-conscious strategy for integrating olfactory testing into routine care, although prospective validation is needed to determine its impact on diagnostic accuracy, referral efficiency, clinical outcomes, and cost-effectiveness.
