# Supplementary material for: Point-of-care smell testing for evaluation of olfactory function: a narrative review of clinical utility
Source: Front Allergy. 2026 Jun 26;7:1882615. doi: 10.3389/falgy.2026.1882615 (PMC13349926; doi:10.3389/falgy.2026.1882615)
Supplement: Supplementary file 1 [file Table1.docx]

**Supplementary table 3-**

**Catalog of Clinical Olfactory Tests: Year Introduced and Manufacturer.**

The table lists each test, the year it was first described or commercialized, and the manufacturer (when known). Trademark designations are preserved (®, ™). Blank company fields indicate that a commercial provider was not specified in the source.

| **Test Name** | **Year Introduced, Company** |
| --- | --- |
| Ascending Methods of Limits (AML) | 1860 |
| Sniff Magnitude Test (SMT) | 1888, CompuSniff, LLC |
| Snap & Sniff® | 1970, Sensonics International |
| Digital Olfactory Testing System (DOTS) | 1982 |
| Smell Identification Test (SIT™) | 1984, Sensonics International |
| 1967 Olfactory Test by Henkin | 1967 |
| Olfactory Perception Threshold Test (OPTT) | 1970 |
| Sniff Bubble | 1970 |
| Asian T and T Test (T&T Test) | 1975 |
| Japanese T&T Olfactometer | 1975 |
| Odor Identification Test | 1984 |
| Olfactory Test | 1984 |
| Smell Awareness Test | 1984 |
| University of Pennsylvania Smell Identification Test (UPSIT®) | 1984, Sensonics International |
| Japanese Open Essence | 1989 |
| Connecticut Chemosensory Clinical Research Center (CCCRC) Test | 1990 |
| Quick Olfactory Sniffin' Sticks Test (Q-Sticks) | 1995 |
| Cross-Cultural Smell Identification Test (CCSIT) | 1996, Sensonics International |
| Alcohol Sniff Test (AST) | 1997 |
| Sniffin' Sticks | 1997, Burghardt® |
| Affective Importance of Odor Scale (AIO) | 1999 |
| Smell Diskettes Olfaction Test (SDOT) | 1999 |
| Retronasal Olfactory Test | 2002 |
| European Test of Olfactory Capabilities | 2003 |
| OSIT-Japanese Odor Stick Test | 2003 |
| Barcelona Smell Test-24 (BAST-24) | 2005 |
| The Questionnaire of Olfactory Disorders (QOD) | 2005 |
| Sino-Nasal Outcome Test (SNOT-22) | 2006 |
| Odor Awareness Scale (OAS) | 2008 |
| Candy Smell Test (CST) | 2011 |
| Pocket Smell Test® (PST) | 2011, Sensonics International |
| NIH Odor Identification Toolbox | 2012 |
| Jet Stream Olfactometry (JSO) | 2014 |
| Self-administered Olfactory Testing System | 2015 |
| Smell-Sensitivity and Smell-Resolution Odor Test | 2017 |
| Indian Smell Test in COVID-19 by AIIMS | 2019 |
| Adaptive Olfactory Measure of Threshold (ArOMa-T) | 2020 |
| SCENTinel® | 2020, Ahersla Health |
| Pediatric Barcelona Olfactory Test-6 (pBOT-6) | 1970 |
| Sniffin’ Kids Test | 1997, Burghardt® |
| Pediatric Smell Wheel™ | 2012, Sensonics International |
| Universal Sniff Test (U-Sniff) | 2018 |
