# Supplementary material for: Point-of-care smell testing for evaluation of olfactory function: a narrative review of clinical utility
Source: Front Allergy. 2026 Jun 26;7:1882615. doi: 10.3389/falgy.2026.1882615 (PMC13349926; doi:10.3389/falgy.2026.1882615)
Supplement: Supplementary file 2 [file Table2.docx]

Supplementary Table 1

**Title of Manuscript:** Olfactory Tests to Evaluate Smell Function at the POC: A Narrative Review of Clinical Utility

**Database:** PubMed/MEDLINE
**Platform:** US National Library of Medicine
**Date Searched:** July 3, 2024 **Search Updated:** December 2024

**Database Date Coverage:** 1946–present
**Date Limits Used:** 2014­–2024

**Other Limits Used:** Language: English; Search strategies used to exclude animal studies, specific publication types, and irrelevant prespecified topics

| **Set** | **Concept** | **Search Strategy** |
| --- | --- | --- |
| #1 | Smell Tests | ("Smell* test*"[Title/Abstract] OR "smell test"[Title/Abstract:~4] OR "smell tests"[Title/Abstract:~4] OR "smell testing"[Title/Abstract:~4] OR "olfact* test*"[Title/Abstract] OR "odor test"[Title/Abstract:~4] OR "odor tests"[Title/Abstract:~4] OR "odor testing"[Title/Abstract:~4] OR "gustat* test*"[Title/Abstract] OR "tast* test*"[Title/Abstract] OR "sniff stick*"[Title/Abstract] OR "sniffin* stick*"[Title/Abstract] OR "smell stick"[Title/Abstract:~0] OR "sniff test"[Title/Abstract:~4] OR "sniff tests"[Title/Abstract:~4] OR "sniff testing"[Title/Abstract:~4] OR "smell wheel"[Title/Abstract] OR "odorant test"[Title/Abstract:~4] OR "u sniff"[Title/Abstract] OR "BAST 24"[Title/Abstract] OR BAST-24[Title/Abstract] OR "odor stick*"[Title/Abstract] OR "Q stick*"[Title/Abstract] OR Q-stick*[Title/Abstract] OR "Monell flavor"[Title/Abstract:~0] OR "snap sniff"[Title/Abstract:~2] OR UPSIT[Title/Abstract] OR SCENTinel[Title/Abstract] OR Scentific[Title/Abstract] OR "T&T olfactometer"[Title/Abstract] OR "scratch and sniff"[Title/Abstract] OR "University of Pennsylvania Smell Identification Test"[Title/Abstract]) |
| #2 | Limit: English Language and Publication Year | #1 AND English[Language] AND ("2014/01/01"[Date - Publication] : "2024/12/31"[Date - Publication]) |
| #3 | Limit: Exclude Animal Studies | #2 NOT ("Animals"[Mesh] NOT ("Animals"[Mesh] AND "Humans"[Mesh])) NOT (mice[Title/Abstract] OR mouse[Title/Abstract] OR murine*[Title/Abstract] OR rat[Title/Abstract] OR rats[Title/Abstract] OR rodent*[Title/Abstract] OR dog[Title/Abstract] OR dogs[Title/Abstract] OR pig[Title/Abstract] OR pigs[Title/Abstract] OR piglet*[Title/Abstract] OR swine[Title/Abstract] OR porcine*[Title/Abstract] OR animal*[Title/Abstract] OR ape[Title/Abstract] OR apes[Title/Abstract] OR monkey*[Title/Abstract] OR gorilla*[Title/Abstract] OR chimpanzee*[Title/Abstract] OR macaque*[Title/Abstract] OR orangutan*[Title/Abstract] OR pongo[Title/Abstract] OR macaca[Title/Abstract] OR "Pan paniscus"[Mesh] OR "Pongo"[Mesh] OR "Macaca"[Mesh] OR "Gorilla gorilla"[Mesh] OR Dogs[Mesh] OR Swine[Mesh] OR Mice[Mesh] OR Rats[Mesh] OR "Muridae"[Mesh] OR "Murinae"[Mesh] OR Rodentia[Mesh] OR "Models, Animal"[Mesh] OR "Animal Experimentation"[Mesh]) |
| #4 | Limit: Exclude Specific Publication Types | #3 NOT (Letter[Publication Type] OR Editorial[Publication Type] OR Comment[Publication Type] OR News[Publication Type] OR "Congress"[Publication Type] OR "Consensus Development Conference"[Publication Type] OR editorial[Title/Abstract] OR commentary[Title/Abstract] OR "conference abstract*"[Title/Abstract] OR "conference proceeding*"[Title/Abstract] OR symposium*[Title/Abstract] OR "Published Erratum"[Publication Type] OR errata[Title/Abstract] OR erratum[Title/Abstract] OR corrigenda[Title/Abstract] OR corrigendum[Title/Abstract] OR protocol[Title] OR protocols[Title] OR "meta-analysis"[Title/Abstract] OR "meta-analyses"[Title/Abstract] OR metanalyses[Title/Abstract] OR metanalysis[Title/Abstract] OR metaanalyses[Title/Abstract] OR metaanalysis[Title/Abstract] OR "meta analyses"[Title/Abstract] OR "meta analysis"[Title/Abstract] OR "Review"[Publication Type] OR "systematic review*"[Title/Abstract] OR "Systematic Review"[Publication Type] OR "Meta-Analysis"[Publication Type] OR "Network Meta-Analysis"[Mesh] OR "integrative review"[Title/Abstract] OR "scoping review"[Title/Abstract] OR "Systematic Reviews as Topic"[Mesh] OR Study Guide[Publication Type]) |
| #5 | Limit: Exclude Irrelevant Topics | #4 NOT (ferment*[Title/Abstract] OR kombucha[Title/Abstract] OR biomass[Title/Abstract] OR farm*[Title/Abstract] OR “bed bug*”[Title/Abstract] OR insecticide*[Title/Abstract] OR cattle[Title/Abstract] OR cows[Title/Abstract] OR cow[Title/Abstract] OR Bovine[Title/Abstract] OR mosquito*[Title/Abstract] OR Anopheles[Title/Abstract] OR plants[Title/Abstract] OR sewer*[Title/Abstract] OR sewage[Title/Abstract] OR Drosophila[Title/Abstract] OR manure[Title/Abstract] OR biowaste[Title/Abstract] OR compost*[Title/Abstract] OR septic[Title/Abstract] OR water[Title/Abstract] OR Manure[Mesh] OR "Sewage"[Mesh] OR "Culicidae"[Mesh] OR "Fermentation"[Mesh] OR "Composting"[Mesh] OR "Bedbugs"[Mesh] OR "Agriculture"[Mesh]) |

**Notes:** The limits for publication year (2014–2024) and language (English) were applied to the main search using the filters available in PubMed. The keywords were searched in the title and abstract fields in PubMed (i.e., [Title/Abstract]), and the controlled vocabulary terms are indicated with [Mesh] or if the MeSH term was not exploded to automatically include all narrower terms this was indicated with [Mesh:Noexp]. Phrases were enclosed in double quotation marks to force the searching of the exact terms in order presented. Proximity searching was used to capture multiple terms appearing in any order within a specified distance of one another in the title and abstract fields (i.e., [Title/Abstract:~4]). Search strategies were used to exclude specific publication types and animal studies as specified in the exclusion criteria. Additionally, a search strategy (#5) was used to exclude irrelevant topics retrieved due to the specific search terms used.
